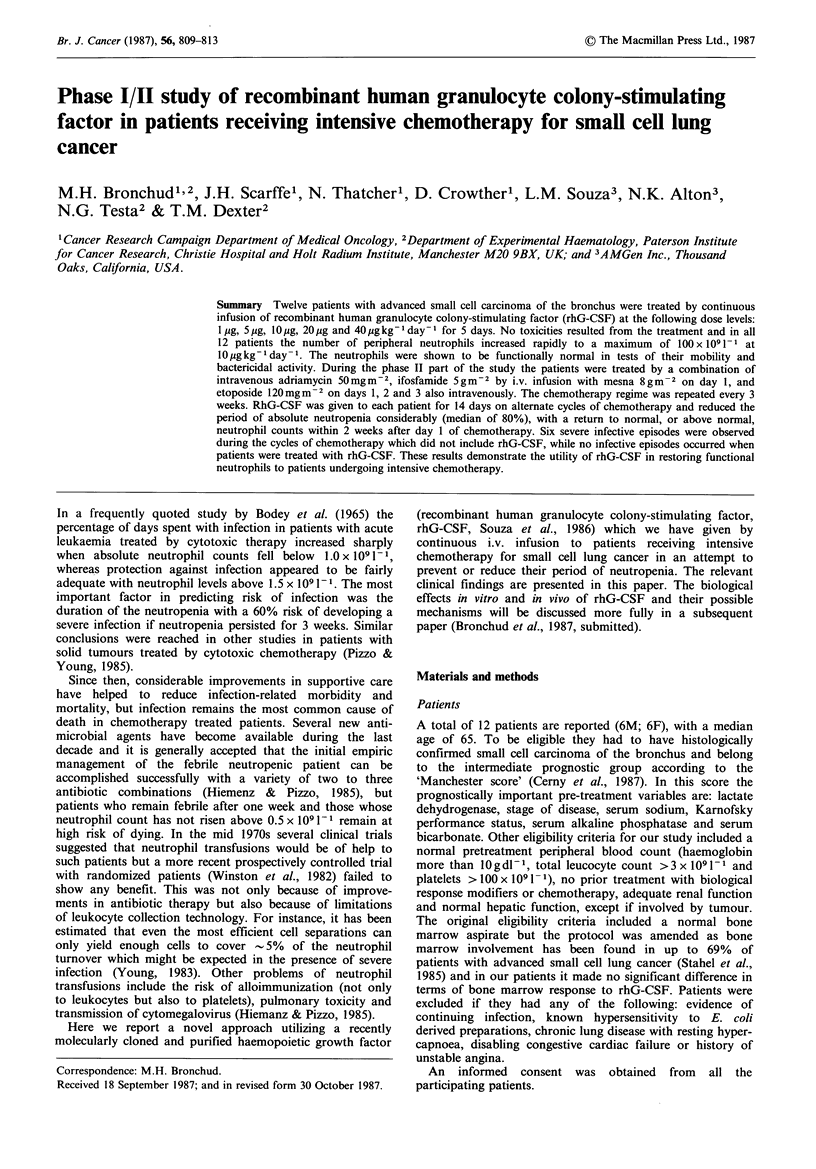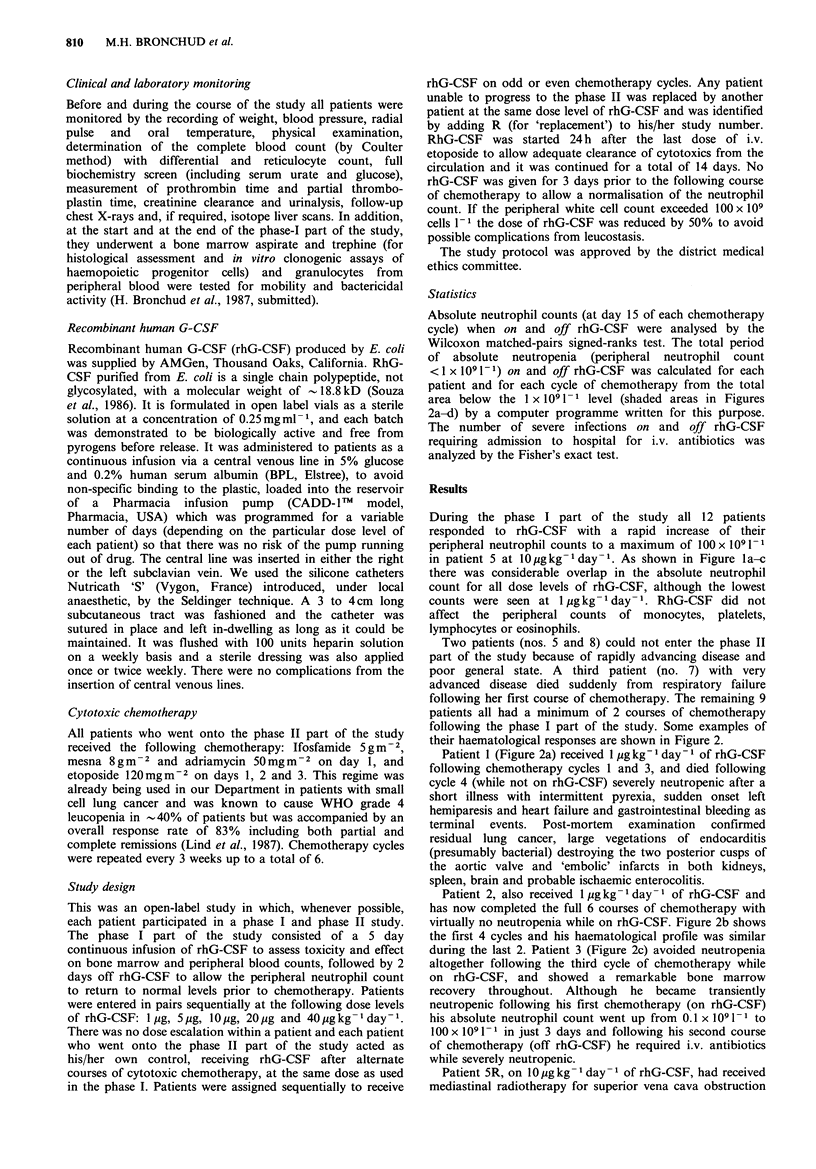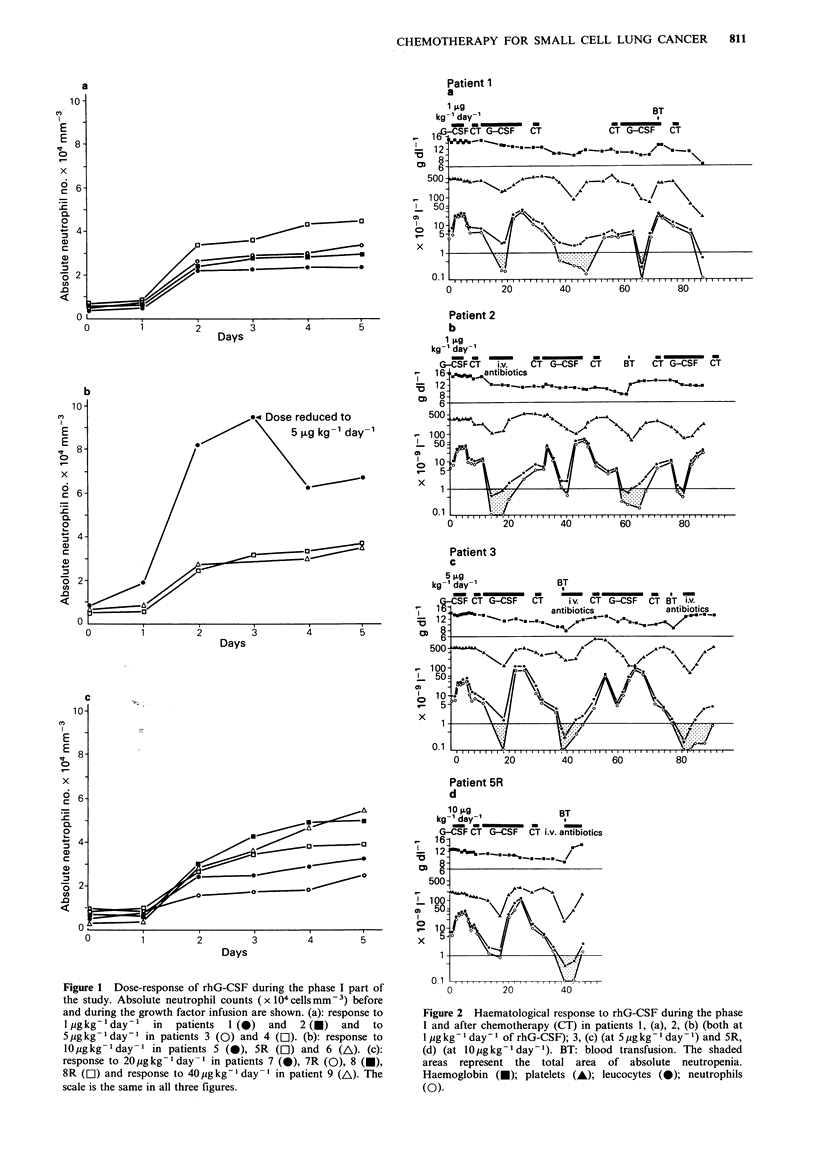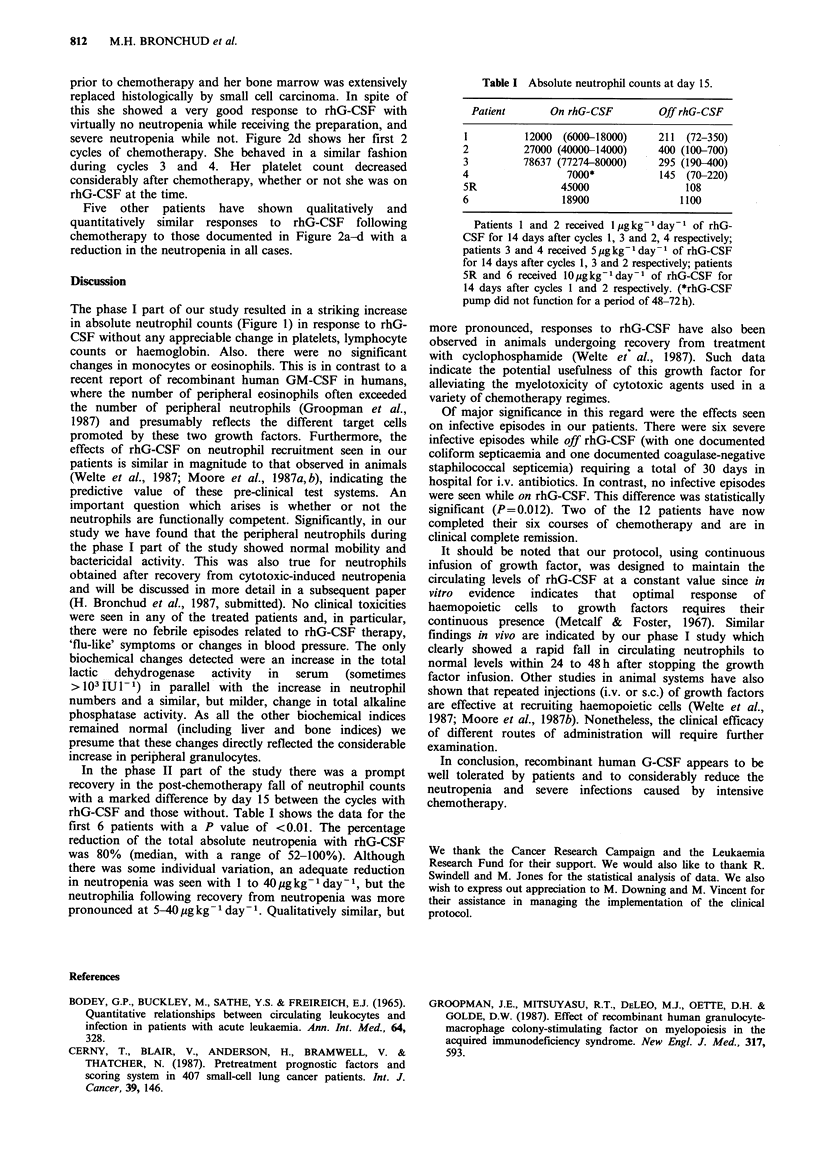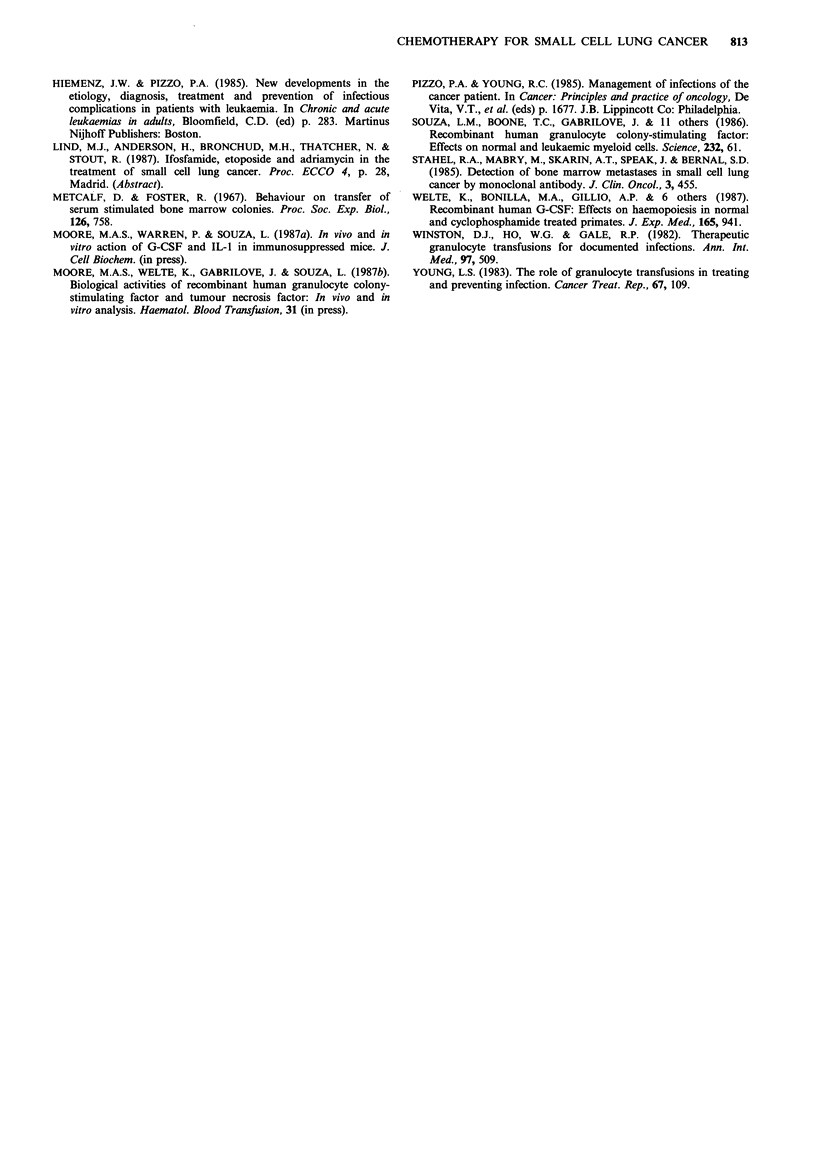# Phase I/II study of recombinant human granulocyte colony-stimulating factor in patients receiving intensive chemotherapy for small cell lung cancer.

**DOI:** 10.1038/bjc.1987.295

**Published:** 1987-12

**Authors:** M. H. Bronchud, J. H. Scarffe, N. Thatcher, D. Crowther, L. M. Souza, N. K. Alton, N. G. Testa, T. M. Dexter

**Affiliations:** Department of Medical Oncology, Paterson Institute for Cancer Research, Christie Hospital and Holt Radium Institute, Manchester, UK.

## Abstract

Twelve patients with advanced small cell carcinoma of the bronchus were treated by continuous infusion of recombinant human granulocyte colony-stimulating factor (rhG-CSF) at the following dose levels: 1 microgram, 5 micrograms, 10 micrograms, 20 micrograms and 40 micrograms kg-1 day-1 for 5 days. No toxicities resulted from the treatment and in all 12 patients the number of peripheral neutrophils increased rapidly to a maximum of 100 x 10(9) l-1 at 10 micrograms kg-1 day-1. The neutrophils were shown to be functionally normal in tests of their mobility and bactericidal activity. During the phase II part of the study the patients were treated by a combination of intravenous adriamycin 50 mg m-2, ifosfamide 5 g m-2 by i.v. infusion with mesna 8 g m-2 on day 1, and etoposide 120 mg m-2 on days 1, 2 and 3 also intravenously. The chemotherapy regime was repeated every 3 weeks. RhG-CSF was given to each patient for 14 days on alternate cycles of chemotherapy and reduced the period of absolute neutropenia considerably (median of 80%), with a return to normal, or above normal, neutrophil counts within 2 weeks after day 1 of chemotherapy. Six severe infective episodes were observed during the cycles of chemotherapy which did not include rhG-CSF, while no infective episodes occurred when patients were treated with rhG-CSF. These results demonstrate the utility of rhG-CSF in restoring functional neutrophils to patients undergoing intensive chemotherapy.


Br. J. Cancer (1987), 56, 809–813 © The Macmillan Press Ltd., 1987

### Phase I/II study of recombinant human granulocyte colony-stimulating factor in patients receiving intensive chemotherapy for small cell lung cancer

M.H. Bronchud[1,2], J.H. Scarffe[1], N. Thatcher[1], D. Crowther[1], L.M. Souza[3], N.K. Alton[3], N.G. Testa[2] & T.M. Dexter[2]

[1]*Cancer Research Campaign Department of Medical Oncology,* [2]*Department of Experimental Haematology, Paterson Institute for Cancer Research, Christie Hospital and Holt Radium Institute, Manchester M20 9BX, UK; and* [3]*AMGen Inc., Thousand Oaks, California, USA.*

**Summary** Twelve patients with advanced small cell carcinoma of the bronchus were treated by continuous infusion of recombinant human granulocyte colony-stimulating factor (rhG-CSF) at the following dose levels: $1\,\mu g$, $5\,\mu g$, $10\,\mu g$, $20\,\mu g$ and $40\,\mu g\,kg^{-1}\,day^{-1}$ for 5 days. No toxicities resulted from the treatment and in all 12 patients the number of peripheral neutrophils increased rapidly to a maximum of $100 \times 10^9\,l^{-1}$ at $10\,\mu g\,kg^{-1}\,day^{-1}$. The neutrophils were shown to be functionally normal in tests of their mobility and bactericidal activity. During the phase II part of the study the patients were treated by a combination of intravenous adriamycin $50\,mg\,m^{-2}$, ifosfamide $5\,g\,m^{-2}$ by i.v. infusion with mesna $8\,g\,m^{-2}$ on day 1, and etoposide $120\,mg\,m^{-2}$ on days 1, 2 and 3 also intravenously. The chemotherapy regime was repeated every 3 weeks. RhG-CSF was given to each patient for 14 days on alternate cycles of chemotherapy and reduced the period of absolute neutropenia considerably (median of 80%), with a return to normal, or above normal, neutrophil counts within 2 weeks after day 1 of chemotherapy. Six severe infective episodes were observed during the cycles of chemotherapy which did not include rhG-CSF, while no infective episodes occurred when patients were treated with rhG-CSF. These results demonstrate the utility of rhG-CSF in restoring functional neutrophils to patients undergoing intensive chemotherapy.

In a frequently quoted study by Bodey *et al.* (1965) the percentage of days spent with infection in patients with acute leukaemia treated by cytotoxic therapy increased sharply when absolute neutrophil counts fell below $1.0 \times 10^9\,l^{-1}$, whereas protection against infection appeared to be fairly adequate with neutrophil levels above $1.5 \times 10^9\,l^{-1}$. The most important factor in predicting risk of infection was the duration of the neutropenia with a 60% risk of developing a severe infection if neutropenia persisted for 3 weeks. Similar conclusions were reached in other studies in patients with solid tumours treated by cytotoxic chemotherapy (Pizzo & Young, 1985).

Since then, considerable improvements in supportive care have helped to reduce infection-related morbidity and mortality, but infection remains the most common cause of death in chemotherapy treated patients. Several new anti-microbial agents have become available during the last decade and it is generally accepted that the initial empiric management of the febrile neutropenic patient can be accomplished successfully with a variety of two to three antibiotic combinations (Hiemenz & Pizzo, 1985), but patients who remain febrile after one week and those whose neutrophil count has not risen above $0.5 \times 10^9\,l^{-1}$ remain at high risk of dying. In the mid 1970s several clinical trials suggested that neutrophil transfusions would be of help to such patients but a more recent prospectively controlled trial with randomized patients (Winston *et al.*, 1982) failed to show any benefit. This was not only because of improvements in antibiotic therapy but also because of limitations of leukocyte collection technology. For instance, it has been estimated that even the most efficient cell separations can only yield enough cells to cover ~5% of the neutrophil turnover which might be expected in the presence of severe infection (Young, 1983). Other problems of neutrophil transfusions include the risk of alloimmunization (not only to leukocytes but also to platelets), pulmonary toxicity and transmission of cytomegalovirus (Hiemanz & Pizzo, 1985).

Here we report a novel approach utilizing a recently molecularly cloned and purified haemopoietic growth factor (recombinant human granulocyte colony-stimulating factor, rhG-CSF, Souza *et al.*, 1986) which we have given by continuous i.v. infusion to patients receiving intensive chemotherapy for small cell lung cancer in an attempt to prevent or reduce their period of neutropenia. The relevant clinical findings are presented in this paper. The biological effects *in vitro* and *in vivo* of rhG-CSF and their possible mechanisms will be discussed more fully in a subsequent paper (Bronchud *et al.*, 1987, submitted).

Correspondence: M.H. Bronchud.
Received 18 September 1987; and in revised form 30 October 1987.

### Materials and methods

### *Patients*

A total of 12 patients are reported (6M; 6F), with a median age of 65. To be eligible they had to have histologically confirmed small cell carcinoma of the bronchus and belong to the intermediate prognostic group according to the 'Manchester score' (Cerny *et al.*, 1987). In this score the prognostically important pre-treatment variables are: lactate dehydrogenase, stage of disease, serum sodium, Karnofsky performance status, serum alkaline phosphatase and serum bicarbonate. Other eligibility criteria for our study included a normal pretreatment peripheral blood count (haemoglobin more than $10\,g\,dl^{-1}$, total leucocyte count $>3 \times 10^9\,l^{-1}$ and platelets $>100 \times 10^9\,l^{-1}$), no prior treatment with biological response modifiers or chemotherapy, adequate renal function and normal hepatic function, except if involved by tumour. The original eligibility criteria included a normal bone marrow aspirate but the protocol was amended as bone marrow involvement has been found in up to 69% of patients with advanced small cell lung cancer (Stahel *et al.*, 1985) and in our patients it made no significant difference in terms of bone marrow response to rhG-CSF. Patients were excluded if they had any of the following: evidence of continuing infection, known hypersensitivity to *E. coli* derived preparations, chronic lung disease with resting hypercapnoea, disabling congestive cardiac failure or history of unstable angina.

An informed consent was obtained from all the participating patients.

*Clinical and laboratory monitoring*

Before and during the course of the study all patients were monitored by the recording of weight, blood pressure, radial pulse and oral temperature, physical examination, determination of the complete blood count (by Coulter method) with differential and reticulocyte count, full biochemistry screen (including serum urate and glucose), measurement of prothrombin time and partial thromboplastin time, creatinine clearance and urinalysis, follow-up chest X-rays and, if required, isotope liver scans. In addition, at the start and at the end of the phase-I part of the study, they underwent a bone marrow aspirate and trephine (for histological assessment and *in vitro* clonogenic assays of haemopoietic progenitor cells) and granulocytes from peripheral blood were tested for mobility and bactericidal activity (H. Bronchud *et al.*, 1987, submitted).

*Recombinant human G-CSF*

Recombinant human G-CSF (rhG-CSF) produced by *E. coli* was supplied by AMGen, Thousand Oaks, California. RhG-CSF purified from *E. coli* is a single chain polypeptide, not glycosylated, with a molecular weight of $\sim$18.8 kD (Souza *et al.*, 1986). It is formulated in open label vials as a sterile solution at a concentration of 0.25 mg ml$^{-1}$, and each batch was demonstrated to be biologically active and free from pyrogens before release. It was administered to patients as a continuous infusion via a central venous line in 5% glucose and 0.2% human serum albumin (BPL, Elstree), to avoid non-specific binding to the plastic, loaded into the reservoir of a Pharmacia infusion pump (CADD-1™ model, Pharmacia, USA) which was programmed for a variable number of days (depending on the particular dose level of each patient) so that there was no risk of the pump running out of drug. The central line was inserted in either the right or the left subclavian vein. We used the silicone catheters Nutricath 'S' (Vygon, France) introduced, under local anaesthetic, by the Seldinger technique. A 3 to 4 cm long subcutaneous tract was fashioned and the catheter was sutured in place and left in-dwelling as long as it could be maintained. It was flushed with 100 units heparin solution on a weekly basis and a sterile dressing was also applied once or twice weekly. There were no complications from the insertion of central venous lines.

*Cytotoxic chemotherapy*

All patients who went onto the phase II part of the study received the following chemotherapy: Ifosfamide 5 g m$^{-2}$, mesna 8 g m$^{-2}$ and adriamycin 50 mg m$^{-2}$ on day 1, and etoposide 120 mg m$^{-2}$ on days 1, 2 and 3. This regime was already being used in our Department in patients with small cell lung cancer and was known to cause WHO grade 4 leucopenia in $\sim$40% of patients but was accompanied by an overall response rate of 83% including both partial and complete remissions (Lind *et al.*, 1987). Chemotherapy cycles were repeated every 3 weeks up to a total of 6.

*Study design*

This was an open-label study in which, whenever possible, each patient participated in a phase I and phase II study. The phase I part of the study consisted of a 5 day continuous infusion of rhG-CSF to assess toxicity and effect on bone marrow and peripheral blood counts, followed by 2 days off rhG-CSF to allow the peripheral neutrophil count to return to normal levels prior to chemotherapy. Patients were entered in pairs sequentially at the following dose levels of rhG-CSF: 1 $\mu$g, 5 $\mu$g, 10 $\mu$g, 20 $\mu$g and 40 $\mu$g kg$^{-1}$ day$^{-1}$. There was no dose escalation within a patient and each patient who went onto the phase II part of the study acted as his/her own control, receiving rhG-CSF after alternate courses of cytotoxic chemotherapy, at the same dose as used in the phase I. Patients were assigned sequentially to receive rhG-CSF on odd or even chemotherapy cycles. Any patient unable to progress to the phase II was replaced by another patient at the same dose level of rhG-CSF and was identified by adding R (for 'replacement') to his/her study number. RhG-CSF was started 24 h after the last dose of i.v. etoposide to allow adequate clearance of cytotoxics from the circulation and it was continued for a total of 14 days. No rhG-CSF was given for 3 days prior to the following course of chemotherapy to allow a normalisation of the neutrophil count. If the peripheral white cell count exceeded $100 \times 10^9$ cells l$^{-1}$ the dose of rhG-CSF was reduced by 50% to avoid possible complications from leucostasis.

The study protocol was approved by the district medical ethics committee.

*Statistics*

Absolute neutrophil counts (at day 15 of each chemotherapy cycle) when *on* and *off* rhG-CSF were analysed by the Wilcoxon matched-pairs signed-ranks test. The total period of absolute neutropenia (peripheral neutrophil count $< 1 \times 10^9$ l$^{-1}$) *on* and *off* rhG-CSF was calculated for each patient and for each cycle of chemotherapy from the total area below the $1 \times 10^9$ l$^{-1}$ level (shaded areas in Figures 2a–d) by a computer programme written for this purpose. The number of severe infections *on* and *off* rhG-CSF requiring admission to hospital for i.v. antibiotics was analyzed by the Fisher's exact test.

### Results

During the phase I part of the study all 12 patients responded to rhG-CSF with a rapid increase of their peripheral neutrophil counts to a maximum of $100 \times 10^9$ l$^{-1}$ in patient 5 at 10 $\mu$g kg$^{-1}$ day$^{-1}$. As shown in Figure 1a–c there was considerable overlap in the absolute neutrophil count for all dose levels of rhG-CSF, although the lowest counts were seen at 1 $\mu$g kg$^{-1}$ day$^{-1}$. RhG-CSF did not affect the peripheral counts of monocytes, platelets, lymphocytes or eosinophils.

Two patients (nos. 5 and 8) could not enter the phase II part of the study because of rapidly advancing disease and poor general state. A third patient (no. 7) with very advanced disease died suddenly from respiratory failure following her first course of chemotherapy. The remaining 9 patients all had a minimum of 2 courses of chemotherapy following the phase I part of the study. Some examples of their haematological responses are shown in Figure 2.

Patient 1 (Figure 2a) received 1 $\mu$g kg$^{-1}$ day$^{-1}$ of rhG-CSF following chemotherapy cycles 1 and 3, and died following cycle 4 (while not on rhG-CSF) severely neutropenic after a short illness with intermittent pyrexia, sudden onset left hemiparesis and heart failure and gastrointestinal bleeding as terminal events. Post-mortem examination confirmed residual lung cancer, large vegetations of endocarditis (presumably bacterial) destroying the two posterior cusps of the aortic valve and 'embolic' infarcts in both kidneys, spleen, brain and probable ischaemic enterocolitis.

Patient 2, also received 1 $\mu$g kg$^{-1}$ day$^{-1}$ of rhG-CSF and has now completed the full 6 courses of chemotherapy with virtually no neutropenia while on rhG-CSF. Figure 2b shows the first 4 cycles and his haematological profile was similar during the last 2. Patient 3 (Figure 2c) avoided neutropenia altogether following the third cycle of chemotherapy while on rhG-CSF, and showed a remarkable bone marrow recovery throughout. Although he became transiently neutropenic following his first chemotherapy (on rhG-CSF) his absolute neutrophil count went up from $0.1 \times 10^9$ l$^{-1}$ to $100 \times 10^9$ l$^{-1}$ in just 3 days and following his second course of chemotherapy (off rhG-CSF) he required i.v. antibiotics while severely neutropenic.

Patient 5R, on 10 $\mu$g kg$^{-1}$ day$^{-1}$ of rhG-CSF, had received mediastinal radiotherapy for superior vena cava obstruction

**Figure 1** Dose-response of rhG-CSF during the phase I part of the study. Absolute neutrophil counts ($\times 10^4$ cells mm$^{-3}$) before and during the growth factor infusion are shown. (a): response to 1 μg kg$^{-1}$ day$^{-1}$ in patients 1 (●) and 2 (■) and to 5 μg kg$^{-1}$ day$^{-1}$ in patients 3 (○) and 4 (□). (b): response to 10 μg kg$^{-1}$ day$^{-1}$ in patients 5 (●), 5R (□) and 6 (△). (c): response to 20 μg kg$^{-1}$ day$^{-1}$ in patients 7 (●), 7R (○), 8 (■), 8R (□) and response to 40 μg kg$^{-1}$ day$^{-1}$ in patient 9 (△). The scale is the same in all three figures.

**Figure 2** Haematological response to rhG-CSF during the phase I and after chemotherapy (CT) in patients 1, (a), 2, (b) (both at 1 μg kg$^{-1}$ day$^{-1}$ of rhG-CSF); 3, (c) (at 5 μg kg$^{-1}$ day$^{-1}$) and 5R, (d) (at 10 μg kg$^{-1}$ day$^{-1}$). BT: blood transfusion. The shaded areas represent the total area of absolute neutropenia. Haemoglobin (■); platelets (▲); leucocytes (●); neutrophils (○).

prior to chemotherapy and her bone marrow was extensively replaced histologically by small cell carcinoma. In spite of this she showed a very good response to rhG-CSF with virtually no neutropenia while receiving the preparation, and severe neutropenia while not. Figure 2d shows her first 2 cycles of chemotherapy. She behaved in a similar fashion during cycles 3 and 4. Her platelet count decreased considerably after chemotherapy, whether or not she was on rhG-CSF at the time.

Five other patients have shown qualitatively and quantitatively similar responses to rhG-CSF following chemotherapy to those documented in Figure 2a–d with a reduction in the neutropenia in all cases.

### Discussion

The phase I part of our study resulted in a striking increase in absolute neutrophil counts (Figure 1) in response to rhG-CSF without any appreciable change in platelets, lymphocyte counts or haemoglobin. Also. there were no significant changes in monocytes or eosinophils. This is in contrast to a recent report of recombinant human GM-CSF in humans, where the number of peripheral eosinophils often exceeded the number of peripheral neutrophils (Groopman *et al.*, 1987) and presumably reflects the different target cells promoted by these two growth factors. Furthermore, the effects of rhG-CSF on neutrophil recruitment seen in our patients is similar in magnitude to that observed in animals (Welte *et al.*, 1987; Moore *et al.*, 1987*a,b*), indicating the predictive value of these pre-clinical test systems. An important question which arises is whether or not the neutrophils are functionally competent. Significantly, in our study we have found that the peripheral neutrophils during the phase I part of the study showed normal mobility and bactericidal activity. This was also true for neutrophils obtained after recovery from cytotoxic-induced neutropenia and will be discussed in more detail in a subsequent paper (H. Bronchud *et al.*, 1987, submitted). No clinical toxicities were seen in any of the treated patients and, in particular, there were no febrile episodes related to rhG-CSF therapy, 'flu-like' symptoms or changes in blood pressure. The only biochemical changes detected were an increase in the total lactic dehydrogenase activity in serum (sometimes $>10^3\,\mathrm{IU\,l^{-1}}$) in parallel with the increase in neutrophil numbers and a similar, but milder, change in total alkaline phosphatase activity. As all the other biochemical indices remained normal (including liver and bone indices) we presume that these changes directly reflected the considerable increase in peripheral granulocytes.

In the phase II part of the study there was a prompt recovery in the post-chemotherapy fall of neutrophil counts with a marked difference by day 15 between the cycles with rhG-CSF and those without. Table I shows the data for the first 6 patients with a $P$ value of $<0.01$. The percentage reduction of the total absolute neutropenia with rhG-CSF was 80% (median, with a range of 52–100%). Although there was some individual variation, an adequate reduction in neutropenia was seen with 1 to $40\,\mu\mathrm{g\,kg^{-1}\,day^{-1}}$, but the neutrophilia following recovery from neutropenia was more pronounced at $5\text{–}40\,\mu\mathrm{g\,kg^{-1}\,day^{-1}}$. Qualitatively similar, but more pronounced, responses to rhG-CSF have also been observed in animals undergoing recovery from treatment with cyclophosphamide (Welte *et al.*, 1987). Such data indicate the potential usefulness of this growth factor for alleviating the myelotoxicity of cytotoxic agents used in a variety of chemotherapy regimes.

**Table I** Absolute neutrophil counts at day 15.

| *Patient* | *On rhG-CSF* | *Off rhG-CSF* |
|---|---|---|
| 1 | 12000 (6000–18000) | 211 (72–350) |
| 2 | 27000 (40000–14000) | 400 (100–700) |
| 3 | 78637 (77274–80000) | 295 (190–400) |
| 4 | 7000* | 145 (70–220) |
| 5R | 45000 | 108 |
| 6 | 18900 | 1100 |

Patients 1 and 2 received $1\,\mu\mathrm{g\,kg^{-1}\,day^{-1}}$ of rhG-CSF for 14 days after cycles 1, 3 and 2, 4 respectively; patients 3 and 4 received $5\,\mu\mathrm{g\,kg^{-1}\,day^{-1}}$ of rhG-CSF for 14 days after cycles 1, 3 and 2 respectively; patients 5R and 6 received $10\,\mu\mathrm{g\,kg^{-1}\,day^{-1}}$ of rhG-CSF for 14 days after cycles 1 and 2 respectively. (*rhG-CSF pump did not function for a period of 48–72 h).

Of major significance in this regard were the effects seen on infective episodes in our patients. There were six severe infective episodes while *off* rhG-CSF (with one documented coliform septicaemia and one documented coagulase-negative staphilococcal septicemia) requiring a total of 30 days in hospital for i.v. antibiotics. In contrast, no infective episodes were seen while *on* rhG-CSF. This difference was statistically significant ($P=0.012$). Two of the 12 patients have now completed their six courses of chemotherapy and are in clinical complete remission.

It should be noted that our protocol, using continuous infusion of growth factor, was designed to maintain the circulating levels of rhG-CSF at a constant value since *in vitro* evidence indicates that optimal response of haemopoietic cells to growth factors requires their continuous presence (Metcalf & Foster, 1967). Similar findings *in vivo* are indicated by our phase I study which clearly showed a rapid fall in circulating neutrophils to normal levels within 24 to 48 h after stopping the growth factor infusion. Other studies in animal systems have also shown that repeated injections (i.v. or s.c.) of growth factors are effective at recruiting haemopoietic cells (Welte *et al.*, 1987; Moore *et al.*, 1987*b*). Nonetheless, the clinical efficacy of different routes of administration will require further examination.

In conclusion, recombinant human G-CSF appears to be well tolerated by patients and to considerably reduce the neutropenia and severe infections caused by intensive chemotherapy.

We thank the Cancer Research Campaign and the Leukaemia Research Fund for their support. We would also like to thank R. Swindell and M. Jones for the statistical analysis of data. We also wish to express out appreciation to M. Downing and M. Vincent for their assistance in managing the implementation of the clinical protocol.

### References

BODEY, G.P., BUCKLEY, M., SATHE, Y.S. & FREIREICH, E.J. (1965). Quantitative relationships between circulating leukocytes and infection in patients with acute leukaemia. *Ann. Int. Med.*, **64**, 328.

CERNY, T., BLAIR, V., ANDERSON, H., BRAMWELL, V. & THATCHER, N. (1987). Pretreatment prognostic factors and scoring system in 407 small-cell lung cancer patients. *Int. J. Cancer*, **39**, 146.

GROOPMAN, J.E., MITSUYASU, R.T., DELEO, M.J., OETTE, D.H. & GOLDE, D.W. (1987). Effect of recombinant human granulocyte-macrophage colony-stimulating factor on myelopoiesis in the acquired immunodeficiency syndrome. *New Engl. J. Med.*, **317**, 593.

HIEMENZ, J.W. & PIZZO, P.A. (1985). New developments in the etiology, diagnosis, treatment and prevention of infectious complications in patients with leukaemia. In *Chronic and acute leukaemias in adults,* Bloomfield, C.D. (ed) p. 283. Martinus Nijhoff Publishers: Boston.

LIND, M.J., ANDERSON, H., BRONCHUD, M.H., THATCHER, N. & STOUT, R. (1987). Ifosfamide, etoposide and adriamycin in the treatment of small cell lung cancer. *Proc. ECCO 4*, p. 28, Madrid. (*Abstract*).

METCALF, D. & FOSTER, R. (1967). Behaviour on transfer of serum stimulated bone marrow colonies. *Proc. Soc. Exp. Biol.*, **126**, 758.

MOORE, M.A.S., WARREN, P. & SOUZA, L. (1987*a*). *In vivo* and *in vitro* action of G-CSF and IL-1 in immunosuppressed mice. *J. Cell Biochem.* (in press).

MOORE, M.A.S., WELTE, K., GABRILOVE, J. & SOUZA, L. (1987*b*). Biological activities of recombinant human granulocyte colony-stimulating factor and tumour necrosis factor: *In vivo* and *in vitro* analysis. *Haematol. Blood Transfusion,* **31** (in press).

PIZZO, P.A. & YOUNG, R.C. (1985). Management of infections of the cancer patient. In *Cancer: Principles and practice of oncology,* De Vita, V.T., *et al.* (eds) p. 1677. J.B. Lippincott Co: Philadelphia.

SOUZA, L.M., BOONE, T.C., GABRILOVE, J. & 11 others (1986). Recombinant human granulocyte colony-stimulating factor: Effects on normal and leukaemic myeloid cells. *Science,* **232,** 61.

STAHEL, R.A., MABRY, M., SKARIN, A.T., SPEAK, J. & BERNAL, S.D. (1985). Detection of bone marrow metastases in small cell lung cancer by monoclonal antibody. *J. Clin. Oncol.*, **3**, 455.

WELTE, K., BONILLA, M.A., GILLIO, A.P. & 6 others (1987). Recombinant human G-CSF: Effects on haemopoiesis in normal and cyclophosphamide treated primates. *J. Exp. Med.*, **165,** 941.

WINSTON, D.J., HO, W.G. & GALE, R.P. (1982). Therapeutic granulocyte transfusions for documented infections. *Ann. Int. Med.*, **97,** 509.

YOUNG, L.S. (1983). The role of granulocyte transfusions in treating and preventing infection. *Cancer Treat. Rep.*, **67,** 109.